# QED cascade saturation in extreme high fields

**DOI:** 10.1038/s41598-018-26785-8

**Published:** 2018-05-30

**Authors:** Wen Luo, Wei-Yuan Liu, Tao Yuan, Min Chen, Ji-Ye Yu, Fei-Yu Li, D. Del Sorbo, C. P. Ridgers, Zheng-Ming Sheng

**Affiliations:** 1 School of Nuclear Science and Technology, University of South China, Hengyang, 421001 China; 2 Key Laboratory for Laser Plasmas (MoE), School of Physics and Astronomy, Shanghai Jiao Tong University, Shanghai, 200240 China; 3 SUPA, Department of Physics, University of Strathclyde, Glasgow, G40NG United Kingdom; 4 IFSA Collaborative Innovation Center, Shanghai Jiao Tong University, Shanghai, 200240 China; 5 York Plasma Institute, Physics Department, University of York, York, YO10 5DQ United Kingdom

## Abstract

Upcoming ultrahigh power lasers at 10 PW level will make it possible to experimentally explore electron-positron (*e*^−^*e*^+^) pair cascades and subsequent relativistic *e*^−^*e*^+^ jets formation, which are supposed to occur in extreme astrophysical environments, such as black holes, pulsars, quasars and gamma-ray bursts. In the latter case it is a long-standing question as to how the relativistic jets are formed and what their temperatures and compositions are. Here we report simulation results of pair cascades in two counter-propagating QED-strong laser fields. A scaling of QED cascade growth with laser intensity is found, showing clear cascade saturation above threshold intensity of ~10^24^ W/cm^2^. QED cascade saturation leads to pair plasma cooling and longitudinal compression along the laser axis, resulting in the subsequent formation of relativistic dense *e*^−^*e*^+^ jets along transverse directions. Such laser-driven QED cascade saturation may open up the opportunity to study energetic astrophysical phenomena in laboratory.

## Introduction

Quantum electrodynamics (QED) cascades (also called avalanches or showers)^1,2^ occur when electrons or positrons radiate hard photons during acceleration or deceleration by strong electromagnetic (EM) fields. These emitted photons may then decay to an electron-positron (*e*^−^*e*^+^) pair in the strong EM fields. The created pairs can emit further photons, which can generate more pairs, and the number of pairs grows exponentially. Cascades initiated by high-energy cosmic rays are responsible for EM showers in the magnetospheres and atmospheres of planets^3^. QED cascades are assumed to be a key mechanism for the production of relativistic *e*^−^*e*^+^ plasmas and jets^4–6^, which are ubiquitous in many extreme astrophysical environments, such as black holes^7^, pulsars^8^, quasars^9^, and are associated with violent emission of short-duration (milliseconds up to a few minutes) gamma-ray bursts^10^. Nevertheless, since the discovery of the relativistic *e*^−^*e*^+^ jets, it has been an unresolved issue on how they are formed and what their temperatures and compositions are^9,11,12^. Reproducing QED cascades and relativistic *e*^−^*e*^+^ jets in the laboratory may significantly enhance our understanding of these energetic astrophysical phenomena. Furthermore, the intense bursts of γ-rays and pairs emitted during QED cascades could find applications in nuclear and particle physics, medical imaging and materials science.

QED cascades will be accessible to upcoming 10 PW-scale laser facilities, such as the Extreme Light Infrastructure (ELI)^13^ and the Exawatt Center for Extreme Light Studies (XCELS)^14^, where the focused laser intensities are expected to reach ~10^23–24^ W/cm^2^. At these intensities, laser-matter interaction enters a new regime characterized by radiation dominated particle dynamics (i.e. dynamics where the radiation reaction force plays an important role)^15–18^, copious *e*^−^*e*^+^ pair production^19–25^ and associated QED cascade development^26–29^ that has attracted significant attention in the last decade^30^. Various EM configurations have been proposed to initiate a cascade of γ-photons and pairs^26–29,31–33^. For example, Bell and Kirk^26^ proposed a configuration composing of two circularly polarized counter-propagating lasers that may induce a QED cascade from seed electrons in the magnetic node. Fedotov *et al*.^27^ investigated the possibility that a single *e*^−^*e*^+^ pair, created by strong laser field in vacuum, would develop an avalanche-like QED cascade, which may occur at threshold intensity of ~10^25^ W/cm^2^. More recently, the growth rate of electron-seeded QED cascades in counter-propagating lasers was studied in the framework of multi-dimensional particle-in-cell (PIC) simulations^33^.

**Figure 1:**
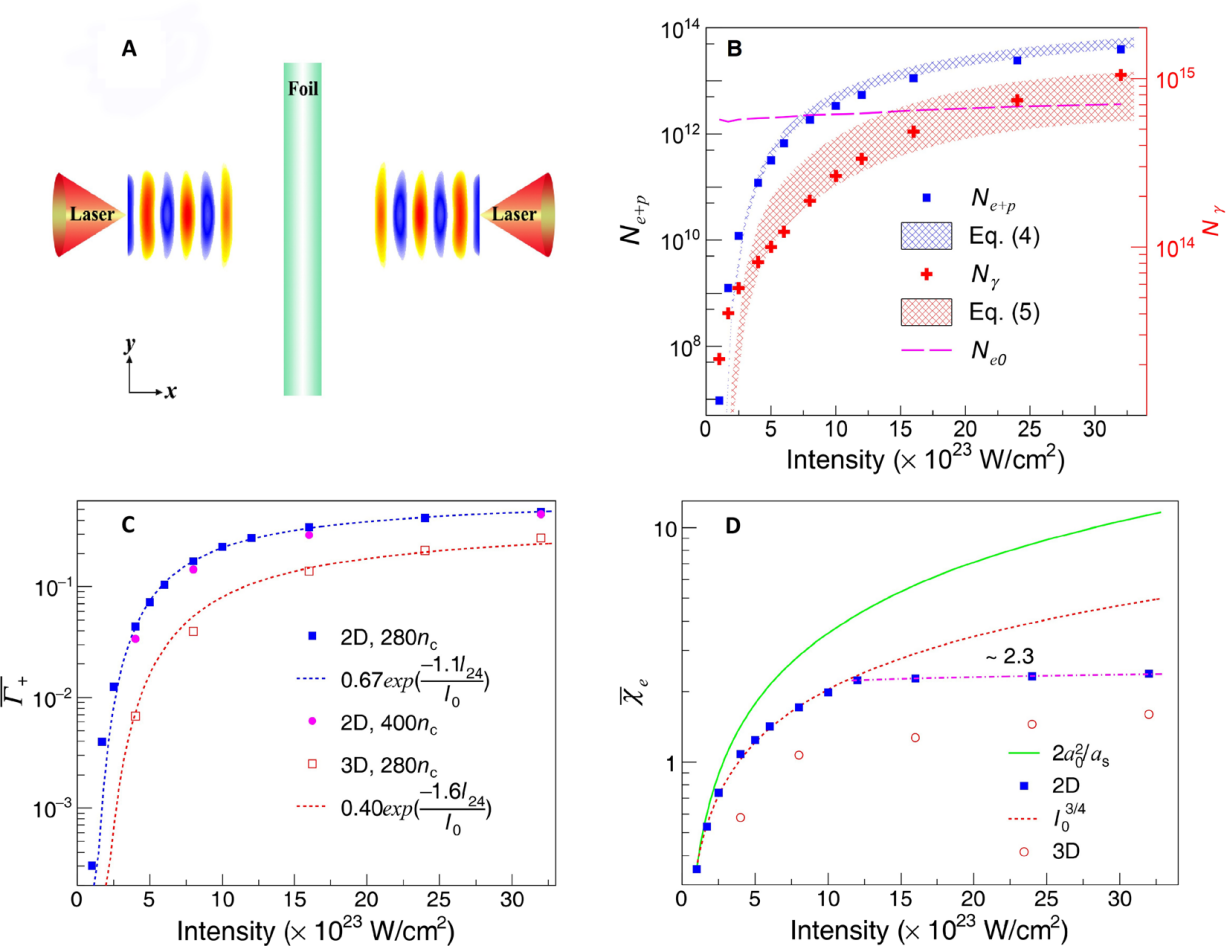
(**A**) Schematic of 2D simulation set-up used to study QED cascade saturation. (**B**) The number of *e*^−^*e*^+^ and γ-photons as a function of laser intensity (*I*_0_) at *t* = 13*T*_0_ (*T*_0_ ≈ 3.3 fs is the laser cycle) in 2D simulations. Only the γ-photons with energy higher than 1.022 MeV are counted. The magenta dashed line shows the estimates of ${N}_{e0}$ at different laser intensities. The blue and red meshed bands indicate the analytical calculations from Eqs (4) and (5) after substituting the scaling function of $\overline{{\Gamma }_{+}}$ in (**C**). The band width is attributed to the variation of ${N}_{e0}$ caused by varying laser intensities from 10^23^ to 3.2 × 10^24^ W/cm^2^. (**C**) Average cascade growth rate $\overline{{\Gamma }_{+}}$ (normalized to *T*_0_) for two different initial plasma densities as a function of laser intensity. The dashed line corresponds to the fitting curve at plasma density of 280*n*_c_ (*n*_*c*_ = ${m}_{e}{\omega }_{l}^{2}/4\pi {e}^{2}$ is the critical plasma density) and ${I}_{24}$ = 10^24^ W/cm^2^. (**D**) Temporally and spatially averaged quantum parameter ${\bar{\chi }}_{e}$ of electrons as a function of laser intensity. The average ${\chi }_{e}$ is obtained by supposing that the produced electrons are located at the antinodes of the electric field. In the absence of two QED processes, one can obtain the maximum ${\chi }_{emax} \sim 2{a}_{0}^{2}/{a}_{s}$^33^, which is shown by the green solid curve for comparison. Here *a*_0_ = $eE/{m}_{e}c{\omega }_{l}$ is the normalized laser field amplitude and *a*_s_ = ${m}_{e}{c}^{2}/\hslash {\omega }_{l}$ the normalized critical field amplitude^35^.

Here we study *e*^−^*e*^+^ cascade saturation and the following nonlinear plasma dynamics with a simple configuration shown in Fig. 1A, where a thin foil is irradiated by two counter-propagating lasers. A scaling law for pair growth is obtained as a function of laser intensity, showing that QED cascade saturation occurs at laser intensities $\gtrsim$10^24^ W/cm^2^. Such cascade saturation results in a dramatic increase of pair plasma density, which causes significant laser energy depletion as the pair plasma becomes opaque to the incident lasers. This finally leads to the emergence of some new high-field phenomena, such as compression of the generated pair plasma and relativistic *e*^−^*e*^+^ jet formation.

## Results

### Laser-driven QED cascade saturation

The rate of an *e*^−^*e*^+^ cascades is determined by the quantum dynamical parameter^34^ ${\chi }_{i}=\,\frac{e\hslash }{{m}_{e}^{3}{c}^{4}}\sqrt{-{({F}_{uv}{p}_{i}^{v})}^{2}}$. Here $i$ refers to the particle species of interest (either electron, positron, or γ-photon); $\hslash$ is the reduced Planck constant; *c* is the speed of light; ${F}_{uv}$ is the electromagnetic tensor; ${p}_{i}^{v}$ is the particle’s four-momentum; and *e* and *m*_*e*_ are electron charge and mass, respectively. It can be approximated as ${\chi }_{i}\approx \frac{{\varepsilon }_{i}}{{m}_{e}{c}^{2}}\frac{{F}_{\perp }}{e{E}_{s}}$ in the ultrarelativistic limit, where ${\varepsilon }_{i}$ is the particle’s energy, ${F}_{\perp }$ is the force acting perpendicular to the particle’s direction of motion, and ${E}_{s}={m}_{e}^{2}{c}^{3}/(e\hslash )\,\simeq$ 1.32 × 10^18^ V/m is the critical electric field of QED^35^. In the scenario of laser foil interaction, QED cascades become important if electrons, on acceleration by the EM field $E$ of the laser, are able to emit γ-ray photons with ${\chi }_{\gamma }\gtrsim 1$. This requires ${\chi }_{e}\gtrsim 1$, and since $E\ll {E}_{s}$, QED cascades need to be initiated by ultra-relativistic electrons. In the course of γ-photon emission, we have ${\chi }_{\gamma }\approx {\chi }_{e}-{\chi }_{e}^{^{\prime} }$ and $0 < {\chi }_{\gamma } < {\chi }_{e}$^36^, where ${\chi }_{e}$ and ${\chi }_{e}^{^{\prime} }$ are the dynamical parameters for the electron before and after emission, respectively. In the course of *e*^−^*e*^+^ pair creation, we have ${\chi }_{p}^{^{\prime\prime} }\,\approx {\chi }_{\gamma }-{\chi }_{e}^{^{\prime\prime} }$ and $0 < {\chi }_{p}^{^{\prime\prime} } < {\chi }_{\gamma }$, where ${\chi }_{p}^{^{\prime\prime} }$ and ${\chi }_{e}^{^{\prime\prime} }$ are the dynamical parameters for the created positron and electron, respectively. Then the ${\chi }_{p,e}^{^{\prime\prime} }$ can be significantly smaller than the ${\chi }_{e}$ and the *e*^−^*e*^+^ pairs are produced with low energies. However, they can be accelerated to high energies by the strong EM field such that ${\chi }_{p,\,e}^{^{\prime\prime} } \sim {\chi }_{e}$. Consequently, the created particles are able to emit further hard photons and the cascade proceeds.

We begin by studying the development of QED cascades over a wide range of laser intensities with two- and three-dimensional (2D and 3D) QED-PIC (particle-in-cell) simulations. The dependence of *e*^−^*e*^+^ and γ-ray yields on laser intensity (${I}_{0}$) is summarized in Fig. 1B. We see that the number of cascade particles grows rapidly as the laser intensity reaches a few 10^23^ W/cm^2^. The rapid increase is replaced by much slower growth when ${I}_{0}\,\gtrsim$ 10^24^ W/cm^2^. According to the analysis of cascade particle dynamics^27,37^, we have developed an analytical model (see Methods) to describe the QED cascade and possible saturation effect in laser foil interactions. The dependence of $\overline{{\Gamma }_{+}}$ on laser intensity is shown in Fig. 1C. In 2D simulations the scaling fits well with

1 $$\overline{{\Gamma }_{+}}\,\simeq \,0.67\exp (\,-\,1.1{I}_{24}/{I}_{0}).$$

Note that $\overline{{\Gamma }_{+}}$ is exponentially small in the quasi-classical limit for ${I}_{0}$ < 10^23^ W/cm^2^, indicating insignificant QED effect. The $\overline{{\Gamma }_{+}}$ value reaches a saturation value when increasing the ${I}_{0}$ to a few times ${I}_{24}$. This suggests that the development of QED cascades approaches saturation and the exponential growth in particle number is quenched. Simulation results shown in Fig. 1B demonstrate this trend. We further compare the $\overline{{\Gamma }_{+}}$ in the simulations with analytical calculations and recover the simulation results (see Fig. 1B), which demonstrates that the scaling formula Eq. (1) works well when describing the development of QED cascades. It is shown in Fig. 1C that the $\overline{{\Gamma }_{+}}$ is insentitive to the initial foil plasma density, generalizing this scaling law.

The saturation of $\overline{{\Gamma }_{+}}$ and the number of *e*^−^*e*^+^ pairs against laser intensity can be interpreted through the dynamical parameter ${\chi }_{e}$ for electrons in the plasma since this plays a controlling role in the development of the QED cascade. The parameter ${\bar{\chi }}_{e}$ averaged in space and time over the QED cascade as a function of laser intensity is shown in Fig. 1D. In the weak-field regime, the ${\bar{\chi }}_{e}$ is approximately equal to its maximum scaling^33^ ${\chi }_{emax} \sim 2{a}_{0}^{2}/{a}_{s}$, implying insignificant QED cascades. As laser intensity reaches a few 10^23^ W/cm^2^, the scaling for the average quantum parameter obtained in 2D simulations is strongly modified to ${\bar{\chi }}_{e}\propto {I}_{0}^{3/4}$. As discussed by Zhang *et al*.^38^, considering the case of a standing wave set up by circularly polarised lasers, this is due to radiation reaction, which produces two effects: (1) it limits the scaling of the average Lorentz factor of the electrons and positrons with ${a}_{0}$ to ${a}_{0}^{1/4}{{\bar{\chi }}_{e}}^{1/6}$, and (ii) it causes the electric field of the laser to no longer be perpendicular to the electron and positron motion (circular for the case of circularly polarised lasers). For the latter case, a factor $\sin \,\theta =\gamma /a$ is introduced into the scaling for ${\bar{\chi }}_{e}$. Here $\theta$ is the angle between the electric field of the laser and the momentum of the electron or positron with Lorentz factor $\gamma$, and $a$ is the transient amplitude of the laser field. Note that a modified classical treatment of radiation reaction has been used to give the scaling for the average Lorentz factor in the strong radiation-damping limit mentioned above. This modified classical treatment includes the reduction in the radiated power due to quantum effects but not the stochasticity of the emission process. Ridgers *et al*.^39^ and Niel *et al*.^40^ recently demonstrated that this is sufficient for predicting average quantities such as ${\bar{\chi }}_{e}.$ The transition between the weak and strong radiation-damping scalings can clearly be seen in Fig. 1D (despite the fact that we have simulated linearly polarised laser pulses). A final change to the scaling of ${\bar{\chi }}_{e}\,\,$with laser intensity occurs when the intensity is sufficient to initiate a strong cascade. In this phase a considerable amount of laser energy is converted into *e*^−^*e*^+^ pairs and *γ*-photons (Supplementary Section S1), and the particle number increases dramatically. ${\bar{\chi }}_{e}$ value finally stops rising and remains nearly constant at 2.3, due to rapid depletion of the incoming laser pulses. As a consequence, the number of *e*^−^*e*^+^ pairs increases only slowly with the laser intensity. Their average Lorentz factor is also found to decrease with the increasing laser intensity.

There is no visible difference between the variation trends of average quantum parameters of the 2D and 3D simulations, although the ${\bar{\chi }}_{e}$ becomes slightly lower in 3D simulations than in 2D simulations (Fig. 1D). In the 3D simulation, due to an additional laser dispersion and plasma expansion along the *z* dimension, particle acceleration in the laser field lasts for a shorter time, in comparison with the 2D case mentioned herein. In addition, the particles created may have a small leak through the additional *z*-axis. Both of these lead to a bit smaller values for both the ${\bar{\chi }}_{e}$ and $\overline{{\Gamma }_{+}}$ (Fig. 1C,D). Therefore, we see that cascade saturation is delayed slightly compared to the 2D case, and the laser energy conversion to *e*^−^*e*^+^ pairs and γ-photons becomes less efficient in the 3D simulation case. It is shown that the laser to particle conversion efficiency is reduced by up to 50–67% when compared to the 2D case (Supplementary Section S1).

The onset of cascade saturation occurs at ${I}_{0}$ ~ 1.1 ${I}_{24}$ and ~1.6 ${I}_{24}$ for two and three dimensions, respectively (Fig. 1C), above which the saturation effect becomes significant. QED cascade saturation leads to highly efficient conversion from laser photons to *e*^−^*e*^+^ pairs (${\eta }_{pair}$) and γ-photons (${\eta }_{\gamma -photon}$) (Supplementary Section S1). At laser intensities exceeding 1.2 ${I}_{24}$, the ${\eta }_{pair}$ obtained in the 2D case is $10 \%$, while the ${\eta }_{\gamma -photon}$ is expected to be $\sim 70 \%$. This results in a positron yield at *I*_0_ = 1.2 ${I}_{24}$ of up to 2.7 × 10^12^, which is enhanced by fifty times compared to that achieved at ${I}_{0}=$ 0.4 ${I}_{24}$. The cascade saturation gives rise to a significant increase of the pair plasma density, and enables new charged particle dynamics to occur, *i.e*. pair plasma compression and consequent formation of relativistic *e*^−^*e*^+^ jets.

### Pair plasma compression and *e*^−^*e*^+^ jet formation

**Figure 2:**
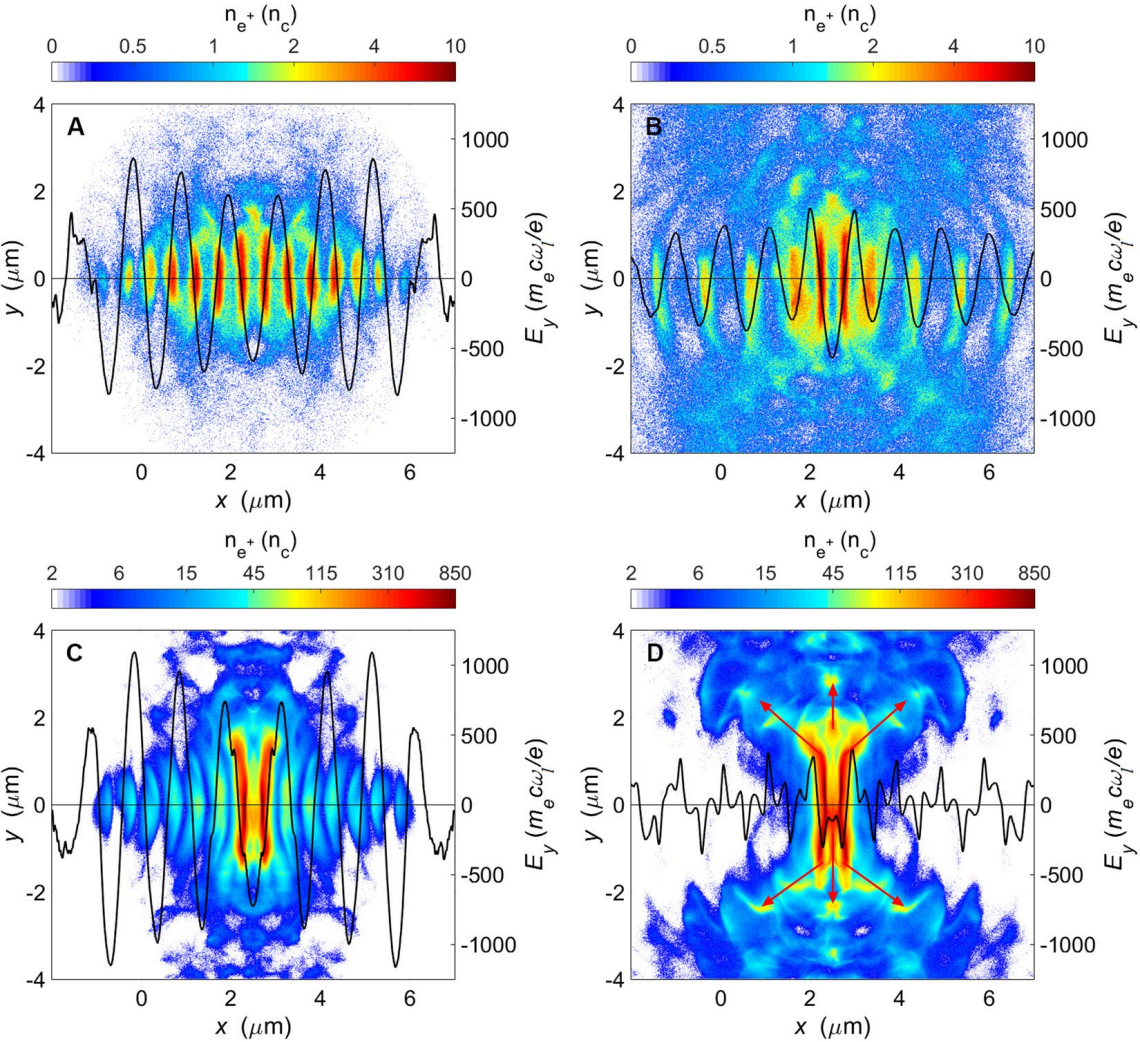
Density maps of the created positrons (contour profile) and longitudinal profiles of the normalized electric fields *E*_*y*_ at *y* = 0 (solid line) at *t* = 10*T*_0_ [(**A,C**)] and *t* = 13*T*_0_ [(**B,D**)]. In (**A** and **B**), the lasers with intensity of 0.4 *I*_24_ are used, and in (**C** and **D**) the laser intensity is 1.2 *I*_24_.

To show the pair plasma compression and relativistic *e*^−^*e*^+^ jet formation we present 2D simulations at laser intensities of ${I}_{0}$ = 0.4 *I*_24_ and 1.2 *I*_24_. The different laser-plasma dynamics of these two cases is shown in Fig. 2 and Supplementary Section S2. At lower intensities, highly relativistic *e*^−^*e*^+^ pairs are able to collide head-on with incoming laser pulses. They emit energetic radiation by nonlinear Compton scattering and therefore lose a considerable amount of their kinetic energy. As the radiation loss continues, these pairs subsequently become trapped in the nodes of the electric field in the standing wave (SW) created by the colliding pulses^17^, coinciding with the locations of the minimum of the ponderomotive potential (Fig. 2A,B). The pairs remain trapped until the laser pulses have passed (Fig. 2B). This plasma dynamics is referred to as normal radiative trapping (NRT)^41^, which has been reported by Chang *et al*.^23^ and Baumann *et al*.^42^.

At higher intensities, charged particle dynamics is remarkably different. Since the created *e*^−^*e*^+^ pairs can be accelerated to higher averaged Lorentz factors, larger values of spatially averaged ${\chi }_{i}$ are obtained accordingly. The trapped *e*^−^*e*^+^ pairs experience much stronger radiation reaction and laser ponderomotive forces, and then they start to migrate away from the electric nodes (Fig. 2C). Such dispersion is favorable to further development of the QED cascade, which in turn enhances the radiation loss. Simulations indicate that the dispersed pairs can lose almost their entire energy within just a few laser periods. For example, at ${I}_{0}$ = 1.2 ${I}_{24}$ each positron is able to emit on average eight hard photons per laser period with average energy of 30 MeV. Strong QED cascades give rise to a continuous increase of the pair plasma density due to efficient laser energy transfer in this system. It is shown that two symmetric high-density layers of positron bunches (Fig. 2C) are formed near the vicinity of *x* = (2.50 ± 0.25)*λ*_*l*_. These high-density layers have a peak density of 10^24^ cm^−3^, exceeding the relativistically corrected critical density ${\bar{\gamma }}_{e}{n}_{c}$ after *t* $\simeq$ 8.75*T*_0_ in this case. Here ${\bar{\gamma }}_{e}$ is the avearge Lorentz factor for the pair plasma. Such dense plasma becomes opaque to the incident laser pulses, which are partially reflected from the high-density layers with a large amount of laser energy absorbed. Consequently, the SW that can be formed at lower intensities does not exist when the pair cascade saturates. The electron and positron bunches, which are located in the nodes furthest from the centre, are compressed inward from two sides by laser ponderomotive forces and pile up around the initial position of the foil (Fig. 2C). During the compression, high-energy *e*^−^*e*^+^ pairs interact with the reflected laser pulses and emit hard photons in their propagation directions (Supplementary Fig. S2), thus developing QED cascades once again. These piled-up pair plasmas are ejected simultaneously along both transverse and longitudinal directions. The evolving relativistic jets finally display multi-polar symmetry patterns in space (see arrows in Fig. 2D).

**Figure 3:**
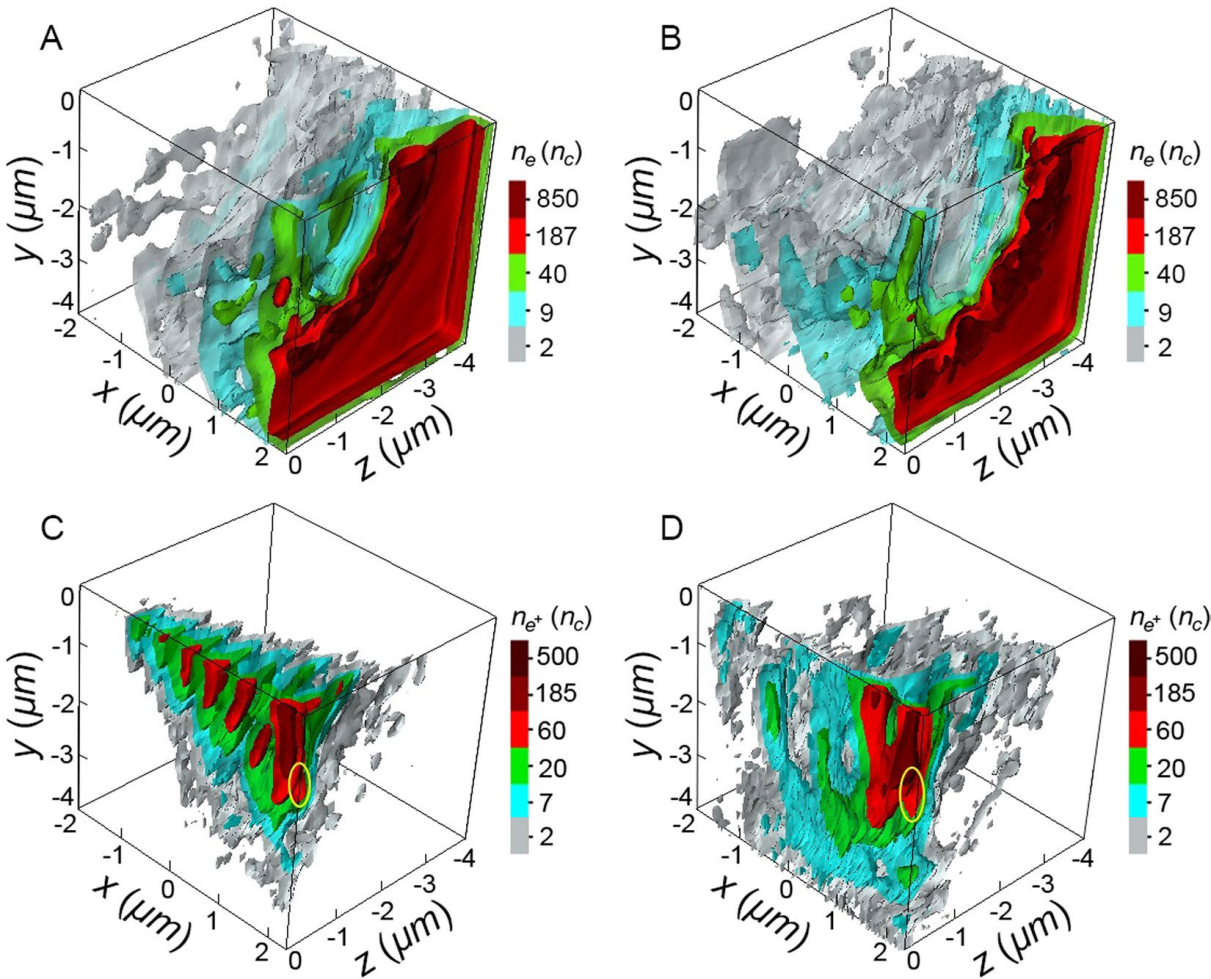
3D contour plots of spatial distributions of foil electrons (upper plots) and created positrons (lower plots) at $t=10{T}_{0}$ [(**A,C**)] and $t=13{T}_{0}$ [(**B,D**)] for the laser intensity of 1.6 ${I}_{24}$. Due to the symmetric structure of particle density along the axes, we only intercept one-eighth part of the cube. The yellow circle lines in lower pads display the positron jets that are ejected simultaneously along the transverse direction.

High-field phenomena such as pair plasma compression and the consequent *e*^−^*e*^+^ jet formation have also been observed in more realistic 3D simulations. The contour distributions of foil electrons (upper plots) and created positrons (lower plots) at the laser intensity of 1.6 ${I}_{24}$ are shown in Fig. 3. It can be seen that foil electrons in the laser focus are expelled both longitudinally and transversely by the strong laser ponderomotive force, and then are accumulated into high-density rings around the hole-boring area (see Fig. 3A,B). The strong QED cascades result in a significant increase of the particle density and the produced pair plasmas can be denser than 500*n*_c_. Such plasma therefore becomes opaque to the incident laser pulses. Consequently, the transient standing wave formed at the early stage by the colliding pulses is destroyed and the laser ponderomotive pressure starts to play a dominant role in the compression of outer layers of the *e*^−^*e*^+^ pairs. The longitudinal compression of the produced *e*^−^*e*^+^ pair plasma along the laser axis is clearly displayed in Fig. 3C,D, where high-density positron layers have migrated from the outer nodes of the electric field towards the initial position of the foil. Such migration is very similar to that observed in the 2D simulations (see Fig. 2C,D). Note that due to an additional plasma expansion along the *z* dimension the density of the compressed electron and positron bunches does not rise as fast as in the 2D case, thus delaying their arrival at center position of the foil. Furthermore, the *e*^−^*e*^+^ jet formation in the laser polarization plane can still be visible, as marked with circle lines in Fig. 3C,D. These results indicate the phenomenologically similar behavior of the post-saturation cascade dynamics in 2D and 3D cases.

The dynamics of pair plasma compression and jet formation happens in the regime of cascade saturation and is due to the increasing influence of the pair plasma as it becomes denser, which leads to the strong absorption of the laser pulse and so the disappearance of the SW fields. In our scheme, the threshold intensity to cause this compression effect is about ${I}_{24}$. Furthermore, additional simulations performed with an initially thick plasma slab (e.g., 5 μm) at near-critical-density suggest that similar laser-plasma dynamics can be observed as long as the laser intensity is above 10^24^ W/cm^2^. We should emphasize that such particle dynamics is different from both NRT^41^ and anomalous radiative trapping (ART)^16^, which demonstrates that particles in very intense SWs are compressed toward, and oscillate synchronously at, the antinodes of the electric field. More recently Efimenko *et al*.^18^ stressed the importance of using ART to produce extreme plasma states in laser-driven *e*-dipole field.

## Discussion

QED cascade saturation leads to strong depletion of the laser energy in the overlapping region of the two pulses, as displayed in Fig. 2D. Highly efficient conversion from laser photons to *e*^−^*e*^+^ pairs and γ-photons occurs (Supplementary Fig. S1). The 2D QED-PIC simulations show the ${\eta }_{pair}$ can reach $10 \%$, which is thirty times higher than that (0.28%) achieved by an alternative scheme where two counter-propagating lasers interact with near critical density plasmas^19^. Meanwhile, the γ-photon yield obtained can approach 10^15^ and the *e*^−^*e*^+^ yield exceeds 10^13^ with peak density of 10^24^ cm^−3^ (see Figs 1B and 2D, respectively), which is comparable to the pair density expected in some astrophysical objects, such as X-ray pulsars^43^. As compared with the recent LWFA-aided scheme^44^, both the *e*^−^*e*^+^ yield and peak density obtained in this scheme are four orders of magnitude higher, although the laser intensity considered here is two orders of magnitude larger. The unique relativistic *e*^−^*e*^+^ jets found in this particular laser intensity regime may open up the opportunity of studying relevant energetic astrophysical phenomena.

Laser-driven QED cascades have recently been shown to strongly modify fundamental plasma physics processes such as relativistic transparency^25,38^ and lead to the harmonics generation^45^ and the quenching of radiation pressure ion acceleration^21^. These effects can significantly change the achievable charged particle energy. The study of QED cascades in the laboratory also opens up the possibility of investigating fundamental strong-field QED effects. Recent work has shown that the helicity of the photons and the electrons & positrons can alter the cascade dynamics, potentially leading to the creation of spin-polarized plasmas^46,47^. Although these effects have not been included here, they provide further motivation for studying these laser-driven cascades.

In conclusion, we have studied the development of QED cascades and subsequent nonlinear phenomena in counter-propagating laser fields. As laser intensity reaches the order of 10^24^ W/cm^2^, QED cascade saturation occurs. Such saturation leads to pair plasma cooling and longitudinal compression along laser axis, subsequently resulting in the formation of relativistic dense *e*^−^*e*^+^ jets along transverse directions. These strong cascade saturation effects and relativistic *e*^−^*e*^+^ jet formation could be tested experimentally with upcoming high-intensity laser facilities such as ELI^13^ and XCELS^14^.

## Methods

### Numerical Modelling

2D and 3D simulations with the QED-PIC code EPOCH^20,48^ were carried out to study QED cascade development. The emission of γ-photons via nonlinear Compton scattering^49^ and the creation of *e*^−^*e*^+^ pairs via multi-photon Breit-Wheeler process^50^ in the strong laser fields were simulated with a Monte-Carlo algorithm^48^. Feedback between the emission processes and the classical macroscopic fields is included as well as quantum corrections to the photon emission. In those simulations two counter-propagating, *p*-polarized laser pulses with identical intensity are focused to a spot radius of *r* = 1 *μ*m. Each pulse has a wavelength of *λ*_*l*_ = 1 *μ*m and a square temporal profile with duration of 9*T*_0_. The laser has a super-Gaussian spatial profile with electric field as *E* ∝ exp (−*y*^5^*/r*^5^). The two lasers are incident from the left and right boundaries of the simulation box at time *t* = 0 and their fronts reach the target at *t* = 4*T*_0_. In 2D simulations, the simulation box has a size of 9*λ*_*l*_ × 8*λ*_*l*_ with symmetry axis at *x* = 2.5*λ*_*l*_. The foil target, composed of carbon ions and protons with the same number density, is placed in the region of *x* = [2*λ*_*l*_, 3*λ*_*l*_] with electron density of *n*_*e*_ = 280*n*_*c*_. The foil is discretized on a spatial grid with the cell size of 10 nm and is represented by 500 macro electrons and 16 macro ions per cell. The 3D simulation box is sampled by 450 cells in the laser propagation direction and 80 cells in each transverse direction, which corresponds to a physical volume of 9*λ*_*l*_ × 8*λ*_*l*_ × 8*λ*_*l*_. 100 macro electrons and 4 macro ions per cell are placed in the plasma region. Other simulation parameters are kept the same as those in 2D simulations.

### Analytical Modelling

Since the cascade kinetic equations^1^, which have been derived to study the cascades initiated by high-energy cosmic rays^3^, cannot be solved analytically in multi-dimensional cases, we use a simple approach to describe the QED cascades in a thin foil irradiated by two counter-propagating laser pulses. This approach is based on analysis of cascade particle dynamics^27,37^. For simplicity, we assume that the number of pairs grows in a time interval much smaller than the laser period. This assumption can be satisfied well in the high-field regime (i.e. ${\chi }_{\gamma }\gtrsim 1$), since the probability of pair production becomes significant. We also neglect the particle displacement between QED events^37^ and particle leakage from the simulation boundary (note that due to the effect of longitudinal compression of pair plasma, it is difficult for the created electrons and the positrons to leak out along laser axis), the temporal evolution of the number of *e*^−^*e*^+^ and γ-photons are given by

2 $$\frac{d{N}_{e+p}}{dt}=2{W}_{pair}{N}_{\gamma },$$

3 $$\frac{d{N}_{\gamma }}{dt}={W}_{\gamma }({N}_{e+p}+{N}_{e0})-{W}_{pair}{N}_{\gamma }.$$

Solving the above equations and substituting the initial conditions, we obtain the expressions for the number of created *e*^−^*e*^+^ and hard photons

4 $${N}_{e+p}\simeq 0.5{N}_{e0}[exp({\Gamma }_{+}t)+exp(\,-\,{\Gamma }_{+}t)]-{N}_{e0},$$

5 $${N}_{\gamma }\simeq \frac{{N}_{e0}{\Gamma }_{+}}{4{W}_{pair}}[exp({\Gamma }_{+}t)-exp(\,-\,{\Gamma }_{+}t)].$$

Here ${N}_{e0}$ is the number of foil electrons in the laser focus, and ${\Gamma }_{+}$ is the cascade growth rate and takes a form

6 $${\Gamma }_{+}=0.5{W}_{pair}(\sqrt{\frac{8{W}_{\gamma }}{{W}_{pair}}+1}-1),$$

where ${W}_{pair}$ and ${W}_{\gamma }$ are probability rates of pair production and photon emission, respectively. Then the average cascade growth $\overline{{\Gamma }_{+}}$ (see Fig. 1C) is obtained by substituting the time-averaged probabilities of pair creation and photon emission, ${\bar{W}}_{pair}$ and ${\bar{W}}_{\gamma }$, which are given by QED-PIC simulations.

The probability of photon emission is always larger than the probability of pair production in the QED cascade. In the regime of strong QED cascades, as the photon carries away a substantial portion of the electron energy and is emitted in the direction of the electron velocity just before emission, we can assume for the sake of simplicity ${\chi }_{\gamma }\simeq {\chi }_{e}\gg 1$ so that the ratio ${W}_{\gamma }/{W}_{pair}\gtrsim 3.8$^37^. This ratio implies that the energy conversion from laser to *e*^−^*e*^+^ pairs should be less than 15–20% (according to the laser energy partition between hard photons and *e*^−^*e*^+^ pairs produced therein), and the cascade growth rate from Eq. (6) should be satisfied as ${\Gamma }_{+}\lesssim 2.3{W}_{pair}$. These two implications could be regarded as the physical constraints of the development of QED cascades in extreme high fields.

### Data availability

The data that support the findings of this study are available from the 10.15129/ddb55407-5ffa-472a-9ada-9bdc3a92ec39.

## Electronic supplementary material

Supplementary material
