# Supplementary material for: QED cascade saturation in extreme high fields
Source: Sci Rep. 2018 May 30;8:8400. doi: 10.1038/s41598-018-26785-8 (PMC5976799; doi:10.1038/s41598-018-26785-8)
Supplement: Supplementary file 1 — Supplementary material [file 41598_2018_26785_MOESM1_ESM.pdf]

Supplemental material for
QED cascade saturation in extreme high fields

Wen Luo, Wei-Yuan Liu, Tao Yuan, Min Chen, Ji-Ye Yu, Fei-Yu Li, D. Del Sorbo, C. P. Ridgers, Zheng-Ming Sheng

This PDF file includes:

Section S1. Laser energy conversion efficiency.

Section S2. Spatiotemporal dynamics of QED cascades.

Fig. S1. Laser energy conversion efficiency to the cascade $e^{-}e^{+}$ pairs, γ-photons, and foil electrons at plasma density of $280n_c$.

Fig. S2. Spatiotemporal evolutions of the positron and hard photon densities at $y = 0$.

Section S1. Laser energy conversion efficiency

The laser energy partition as a function of laser intensity is shown in fig. S1. By increasing laser intensity, the energy conversion efficiency η_{pair} and $\eta_{\gamma-\mathrm{photon}}$ increase rapidly at first and then get saturated. When laser intensities approach the order of 10^{24} W/cm^2, while the η_{pair} obtained with 2D case maintains a level of 10%, the total conversion efficiency η_{total} is expected to be higher than 85%, thus demonstrating strong laser energy depletion in the laser-plasma system. In addition, as the secondary self-induced QED effect plays a dominant role, the energy conversion efficiency $\eta_{\mathrm{foil}e}$ drops from tens percent to a few percent level. Fig. S1 also shows that the laser to particle conversion efficiency is reduced by up to 50%−67% when compared to the 2D case.

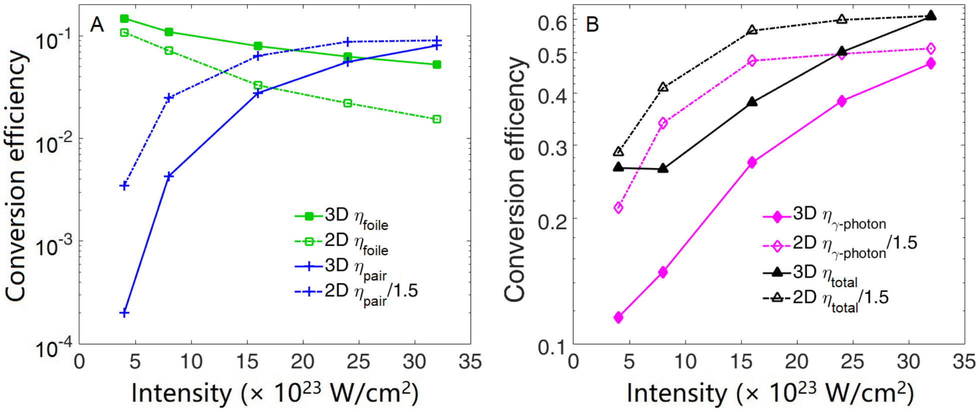

Figure S1. Laser energy conversion efficiency to the foil electrons (η_{foile}), cascade $e^{-}e^{+}$ pairs (η_{pair}), and γ-photons ($\eta_{\gamma-\mathrm{photon}}$) at plasma density of $280n_c$. The η_{total} denotes total energy conversion efficiency to the three kinds of particles. The energy conversion from laser photons to ions could be negligible and is not shown here.

Section S2. Spatiotemporal dynamics of QED cascades

The spatiotemporal dynamics of the QED cascades at $I_0 = 4 \times 10^{23}$ and 1.2×10^{24} W/cm^2 are shown in fig. S2. Bright γ photons, radiated by high-energy foil electrons in the laser focus, propagate outward through the laser fields and interact with the laser waves to produce e^-e^+ pairs via the multi-photon Breit-Wheeler process. For the lower intensity case, these pairs are trapped in the vicinity of the electric field nodes. As the interaction progresses they move outward from the trapping centres when the laser pulses pass their peak amplitude. However, for the higher intensity case the created e^-e^+ pairs from the outer region continuously converge into the inner high-density layers as the result of ponderomotive pressures until $t \sim 13T_0$. During this compression, the dense e^-e^+ pairs collide with the reflected laser pulses, radiating a large number of hard photons, which propagate towards the initial foil position and develop QED cascades once again. At the instant ($t \sim 13T_0$) of vanishing electric fields, the compressed high-density layers fully converge into the centre in the longitudinal direction and then are launched in the transverse directions.

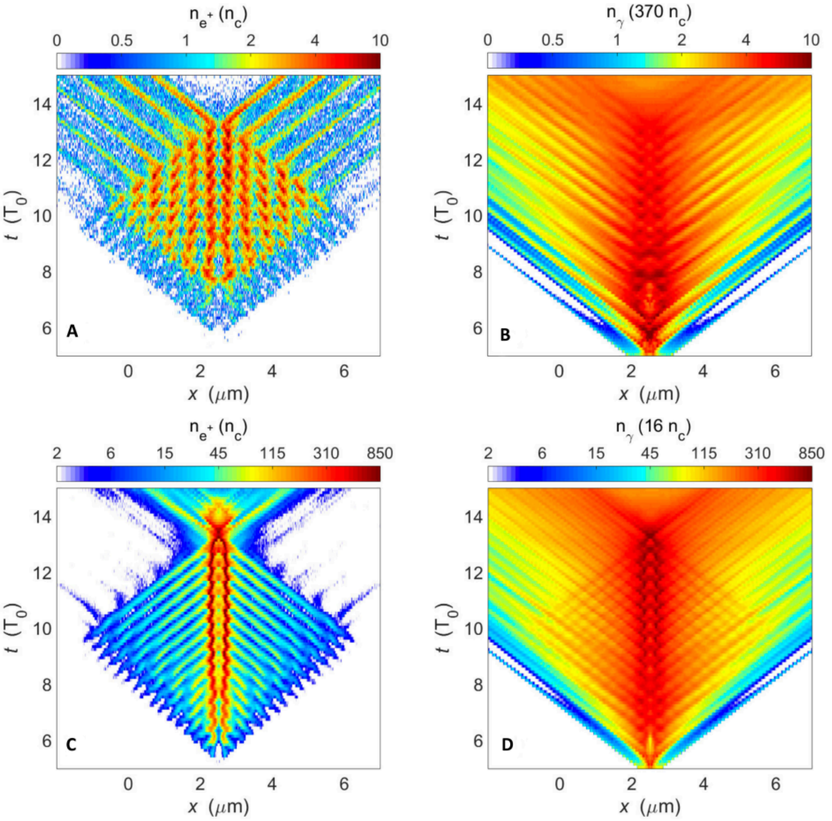

Figure S2. Spatiotemporal evolutions of the positron (**A** and **C**) and hard photon (**B** and **D**) densities at y = 0, where figures (**A** and **B**) and (**C** and **D**) are shown for the cases with the laser intensities of 4×10^{23} and 1.2×10^{24} W/cm^2, respectively. In both cases foil electrons are mainly focused in the electric nodes next to the symmetry axis; the spatiotemporal evolutions of the BW-electron density display similar patterns as these for positrons, thus they are not shown here.
